# A dose planning study for cardiac and lung dose sparing techniques in left breast cancer radiotherapy: Can free breathing helical tomotherapy be considered as an alternative for deep inspiration breath hold?

**DOI:** 10.1016/j.tipsro.2023.100201

**Published:** 2023-01-26

**Authors:** Sara Abdollahi, Mohammad Hadi Hadizadeh Yazdi, Ali Asghar Mowlavi, Sofie Ceberg, Marianne Camille Aznar, Fatemeh Varshoee Tabrizi, Roham Salek, Alireza Ghodsi, Ali Shams

**Affiliations:** a Physics Department, Faculty of Science, Ferdowsi University of Mashhad, Mashhad, Iran; b Medical Physics Department, Reza Radiotherapy and Oncology Center, Mashhad, Iran; c Medical Radiation Physics, Lund University, Lund, Sweden; d Division of Cancer Sciences, School of Medical Sciences, Faculty of Biology, Medicine and Health, University of Manchester, Manchester, United Kingdom; e Radiotherapy and Oncology Department, Reza Radiotherapy and Oncology Center, Mashhad, Iran; f Radiotherapy and Oncology Department, Mashhad University of Medical Science, Mashhad, Iran; g Department of Statistics, Hakim Sabzevari University, Sabzevar, Iran; h Medical Physics Department, Seyed-al-Shohada Hospital, Isfahan, Iran

**Keywords:** Left breast radiotherapy, Deep inspiration breath-hold, Helical Tomotherapy, Toxicity management

## Abstract

**Purpose:**

To investigate the possibility to be able to offer left sided breast cancer patients, not suitable for DIBH, an organ at risk saving treatment.

**Materials and Methods:**

Twenty patients receiving radiotherapy for left breast cancer in DIBH were enrolled in the study. Planning CT scans were acquired in the same supine treatment position in FB and DIBH. 3DCRT_DIBH plans were designed and optimized using two parallel opposed tangent beams (with some additional segments) for the breast and chest wall and anterior-posterior fields for regional lymph nodes irradiation. Additionally, FB helical tomotherapy plans were optimized to minimize heart and lung dose. All forty plans were optimized with at least 95% of the total CTV covered by the 95% of prescribed dose of 50 Gy in 25 fractions.

**Results:**

HT_FB plans showed significantly better dose homogeneity and conformity compared to the 3DCRT_DIBH specially for regional nodal irradiation. The heart mean dose was almost comparable in 3DCRT_DIBH and HT_FB while the volume (%) of the heart receiving 25 Gy had a statistically significant reduction from 7.90 ± 3.33 in 3DCRT_DIBH to 0.88 ± 0.66 in HT_FB. HT_FB was also more effective in left descending artery (LAD) mean dose reduction about 100% from 30.83 ± 9.2 Gy to 9.7 ± 3.1. The ipsilateral lung volume receiving 20 Gy has a further reduction of 43 % in HT_FB compared with 3DCRT_DIBH. For low dose comparison, 3DCRT_DIBH was superior for contralateral organ sparing compared to the HT_FB due to the limited angle for dose delivery.

**Conclusion:**

For patients who cannot be a candidate for DIBH for any reason, HT in free breathing may be a good alternative and provides heart and ipsilateral lung dose sparing, however with the cost of increased dose to contralateral breast and lung.

## Highlights

• Helical Tomotherapy in free breathing; an alternative for heart and ipsilateral lung dose sparing for patient who cannot comply with deep inspiration breath hold.
• Significant contralateral dose reduction with 3D conformal radiotherapy in deep inspiration breath hold compared to Helical Tomotherapy in left breast radiotherapy.
• Helical Tomotherapy; effective for toxicity management for left breast cancer patients with targets with internal mammary and regional lymph nodes.

## Introduction

Radiotherapy is one of the essential modalities of the standard of care in breast cancer treatment after breast-conserving surgery or mastectomy [1], [2], [3], [4], [5], [6], [7]. While this adjuvant treatment approach can improve local control and overall survival, the benefits can be hindered by radiation-induced late side effects, such as cardiovascular disease and second primary cancers [8], [9], [10], [11], [12]. Deep Inspiration Breath Hold (DIBH) radiotherapy has been validated as an effective approach for cardiac and lung dose reduction in left breast radiotherapy [13], [14], [15], [16], [17], [18]. Although most patients can benefit from respiratory management via DIBH, there is still a minority that cannot comply with DIBH and need alternative approaches for toxicity management. Schönecker et al. evaluated the application of DIBH for left-sided breast cancer radiotherapy and reported that 9 out of 13 patients successfully completed the treatment with good compliance and no interruption [19], which means that 31% did not. Al-Hammadi *et al*. evaluated the cardiac sparing of voluntary DIBH for left-sided breast irradiation and stated that nine out of sixty-three patients (14.3%) were excluded at the time of coaching due to non-compliance with the DIBH protocol [20].

Intensity Modulated Radiation Therapy (IMRT) techniques via inverse planning dose calculation methods and step and shoot, rotational or helical dose delivery may be other possible ways to reduce the dose to the heart and ipsilateral lung in left breast irradiation. IMRT can tailor the dose distribution in a concave shape to the left breast and manage the heart and left descending artery (LAD) doses in close proximity to the target volume. However, a larger volume of the heart, spinal cord, contralateral breast, and contralateral lung may be exposed to low-dose irradiation due to the dose delivery from several gantry angles [21], [22], [23], [24], [25]. Helical tomotherapy (HT), as one of the IMRT dose delivery techniques, covers the concave targets with excellent conformity of the helical dose distribution throughout a rotating gantry, binary MLC, and a moving couch. Several studies reported that HT results in significant dosimetric gain related to normal tissue sparing compared to the 3D conformal radiotherapy (3DCRT) technique for breast radiotherapy especially when internal mammary chain and reginal lymph nodes are involved, but it can also lead to larger low-dose regions [26], [27], [28], [29], [30].

Considering local extension of tumors, studies showed that a high proportion of breast cancer patients in Iran (approximately 85%) had advanced stage of disease at their first presentation and at the time of diagnosis [31], [32], [33]. Although recent studies show a decrease in T4 Tumors and increase in early stages detection [34], the majority of cases are still diagnosed with T3 or T2b stages. In recent years, increasing attention has been paid to irradiating the internal mammary lymph nodes (IMN) along with the regional lymph nodes especially for advanced stages of breast cancer to decrease the risk of distant recurrence and improve long-term overall survival [35], [36], [37], [38], [39]. Therefore, the majority of breast radiotherapy patients in Iran are candidate for IMN and regional nodes irradiation, which implies larger and more nonsurface volumes and raises concerns about heart, LAD and lung dose. Our institute treats about 1000 breast cancer patients with radiotherapy per year, out of a total of 3500 patients per year. However, only one of our 5 linacs is equipped with surface guided radiotherapy (SGRT) and motion management technology due to lack of resources. As a result, we need to look for another efficient alternative for toxicity management in breast irradiation. Considering this situation, this study aims to evaluate the efficiency of cardiac and lung sparing techniques with and without DIBH in challenging clinical scenarios in a population more weighted towards high stage patients who had mastectomy surgery and/or IMN treatment.

## Material and methods

A heart-sparing radiotherapy technique incorporating DIBH was implemented for left-breast treatment in Reza Radiotherapy and Oncology Center (RROC), Mashhad, Iran, in 2019.

In the computed tomography (CT) room with Siemens Somatom Definition AS Open scanner (Siemens Medical Solutions, Erlangen, Germany) the surface guided DIBH technique was acquired using Sentinel^TM^ optical surface scanner (C-rad Positioning AB, Uppsala, Sweden). On one of the linacs (Siemens Artiste, Medical Solutions, Erlangen, Germany) patient position verification and intra-fractional motion monitoring of the patients’ surface, including DIBH, was carried out using a commissioned optical surface scanner (Catalyst^TM^, C-rad Positioning AB, Uppsala, Sweden).

### Patient selection

The inclusion criteria for DIBH treatment were: i) the patient is candidate for left breast irradiation, ii) > 10% of the heart volume receives 25 Gy in the 3DCRT free breathing plans and iii) the patient is able to perform four consecutive DIBHs of 25 s each at coaching session. One exclusion criterion was: heart dose was not significantly reduced to meet the dose constraint in DIBH plan for any reason (this decision will be made qualitatively by radiation oncologist and medical physicist based on the best judgment). Twenty-three patients were approached to be included in this study. Both FB and DIBH scans were made for each patient to assess the inclusion criteria. Three patients could not meet the inclusion criteria of DIBH treatment of which two patients could not comply with stable DIBH for 25 s at coaching session, and one patient́s body habitus obscured her chest which made it impossible using the motion management system. Inclusion was stopped when twenty patients were prospectively included in this study. Patient characteristics of this cohort are shown in Table 1. Seventy percent of the patients in this study had mastectomy surgery and/or IMN target. All patients were treated with a conventional fractionated regimen (2 Gy in 25 fractions) and if indicated sequential boost to the tumor bed was applied with a dose of 5 × 2 Gy. The use of the radiotherapy database for this study has been approved by RROC research and education committee and the research ethics committee of the Ferdowsi University of Mashhad (IR.UM.REC.1400.317).

**Table 1:** Patient characteristics.

		Breast/ chest wall only	Regional nodes without IMN	Regional nodes with IMN
Total number of patients	20	1	7	12
Breast conserving surgery	10	1	5	4
Mastectomy	10	–	2	8

### CT simulation

Patients were positioned using Orfit AIO breast and lung board (Orfit Industries NV, Wijnegem, Belgium) with their arms raised over the head and positioned on arm support. They underwent supine CT in free breathing (FB) and DIBH using 3 mm slice thickness. To train the patients to breathe deep in a reproducible way, a coaching session was set for each patient before CT simulation session. Selected patients were given video instruction and information regarding the limitations and benefits of the DIBH technique. Patients had audio-visual feedback (video goggles) in coaching and scanning procedures.

### Treatment planning

The breast clinical target volume (CTV) was delineated according to the Radiation Therapy Oncology Group (RTOG) guidelines [40]. For node positive patients, ipsilateral axillary lymph nodes level II-III and lymph nodes in the supra- and infraclavicular fossa were also included in the CTV. For high-risk patients, IMN was also delineated and added to the CTV. Breast CTV in DIBH was contoured slightly larger than breast CTV in FB with more posterior or inferior extension for some patients. A revision was made to keep the CTVs comparable for both imaging data sets. Planning target volume (PTV) was then defined as a 5 mm margin to the whole CTV and CTV and PTV were retracted 3 mm from the skin surface. Bilateral lungs, contralateral breast, heart, LAD, and spinal canal were outlined as OARs. The heart was delineated from the apex to the inferior border of the left pulmonary artery and included all great vessels except the inferior vena cava [41]. The LAD arteries were delineated using a 6-mm brush considering the motion uncertainties from the left side of the ascending aorta as far as it could be visualized, often to the middle of the heart. To restrict high doses to the PTVs, a ring help structure with 30 mm expansion were created. The spinal cord was also expanded by 3 mm for the spinal cord planning risk volume.

The FB planning CTs were used for HT_FB treatment planning, and DIBH CT was used for 3DCRT_DIBH plans. All patients were treated with 3DCRT_DIBH plans and HT_FB plans were created retrospectively for the purpose of this study. The 3DCRT DIBH plans consisted of two parallel-opposed tangent beams for the breast and chest wall, anterior-posterior fields for regional lymph nodes irradiation and one or two additional segments for each tangent beam to improve the dose homogeneity. The Prowess Panther version 5.5 (Prowess inc., Concord, CA) treatment planning system was used for organ delineation and 3DCRT planning (Collapsed Cone Convolution Superposition with a dose grid resolution of 3 mm × 3 mm × 3 mm). The linac (Siemens Artiste) was equipped with 160 MLC leaves (5 mm thickness at isocenter) and the dose rate was 300MU/min for 6MV photon beam and 500 MU/min for 15MV photon beam.

For Helical Tomotherapy dose delivery (HT_FB), Accuray Precision Radiotherapy Treatment Planning software version V 2.0 (Accuray, Sunnyvale, CA) was used and the image sets and outlined structures were imported from Prowess Panther TPS to the Precision TPS. The delivery machine modeled in TPS is Radixact X9 with a 6MV FFF beam and1000cGy/min dose rate. The pneumatically driven binary MLC with 0.625 cm leaf width projection at isocenter is capable of a minimum leaf opening time of 18 ms. The field width, pitch, and modulation factor parameters were assigned to 5 cm, 0.3/0.43 cm, and 4, respectively and the dynamic jaw mode was selected for all plans. In Precision TPS, all the inverse planning constraints for organs at risk are soft constraints and can be violated, relaxed and tightened during optimization. It also employs a least squares minimization-optimization algorithm and the full dose calculation algorithm is CCCS with pre-computed, Monte Carlo-generated scatter kernels. High resolution gride size of 0.98 mm × 3 mm × 0.98 mm was used for dose calculation. Different “Importance” was set in optimization for each OAR and target as a relative weight to update leaf intensity values with each iteration. The heart was set to have the highest importance among the OARs with almost near to the target’s importance and left lung and ring were set to have a lower importance than heart and higher than contralateral breast and lung and spinal canal. When heart and ipsilateral lung dose constraints were met during the optimization, a fine tuning was done to minimize contralateral organs dose reduction as much as possible while maintaining the target dose coverage. After final dose calculation, when all the clinical objectives and constraints are met, the modulation factor was manually reduced by 0.2 at a time for treatment time reduction until the plan quality starts to degrade or the estimated gantry period reaches 11.8 s. As studies showed that the margin accounting for intrafraction motion in breast radiotherapy is highly dependent on treatment time [42], [43], [44], [45], [46], the beam-on-time reduction was also of attention for HT treatment using optimal combinations of pitch, modulation factor, and field width. However, no target volume margin was considered for baseline shifts or respiratory motions for HT_FB plans. Although directional blocking in helical tomotherapy planning is considered to be effective to reduce the radiation received by contralateral organs [30], no directional blocking was used in the current study as it will compromise the treatment time and introduce more uncertainty to the dose delivery.

To be considered clinically acceptable, all plans needed to cover at least 95% of the total CTV with 95% of the prescribed dose of 50 Gy in 25 fractions. In addition, a qualitative assessment was made by evaluating the dose distributions slice-by-slice to assure of adequate target coverage and OAR sparing for each patient for all plans. The location and magnitude of “hot” and “cold” spots within the target volume were also assessed for each plan. The homogeneity index (HI) for CTV was calculated using D_2_/D_p_ Formula; where D2 is the dose received by 2% of the target volume and Dp is the prescribed dose to the target volume. *V5Gy*, *V*20Gy, *V*40Gy and D_mean_ to the ipsilateral lung; *V5Gy* and D_mean_ to the contralateral lung, *V*25Gy, *V5Gy,* and D_mean_ to the heart, D_mean_ and D2% to the LAD, and mean dose to the contralateral breast were used for the plan comparisons. Table 2. shows the planning objectives which were employed in treatment planning.

**Table 2:** Dose constraints/Planning objectives employed in treatment planning.

Organ at risk	Dose constraint
Heart	Mean < 5 Gy
	V25 < 10%
	V5 < 40%
LAD*	Max < 20 Gy
Ipsilateral lung	V20 < 30%
	Mean < 15 Gy
Contralateral lung	Mean < 5 Gy
Contralateral Breast	Mean < 5 Gy
Spinal Cord	Max < 45 Gy
Ipsilateral Humerus	Max < Prescribed Dose

* LAD max dose constraint was recommended by the Danish Breast Cancer Cooperative Group (DBCG) to be considered to meet when possible [47].

### Planning and delivery time

The treatment planning time was considered as the time from starting a plan until final optimization (for HT inverse planning) and dose calculation are completed. The estimated treatment delivery time for 3DCRT_DIBH was defined as the time from first beam on until the last beam is turned off. The estimated treatment delivery times for HT_FB were calculated and reported by the planning system.

### Statistics

Independent-samples Mann-Whitney *U* test with a significance level of p < 0.05 was used to test for differences between two techniques. All statistical tests were performed in SPSS software (v. 27.0, IBM Corporation, Armonk, NY, USA).

## Results

All HT_FB plans provided acceptable target coverage. For 3DCRT_DIBH technique, the medial part of the supraclavicular fossa target had not fully covered by 95% of the prescribed dose for almost all the patients. A reason could be that the anterior beam was angled for spinal cord sparing and the medial of the CTV was placed at beam penumbra region. The heart mean dose was statistically similar in 3DCRT_DIBH and HT_FB with an average of 5.11 ± 1.55 Gy (mean ± standard deviation) and 4.63 ± 0.79 Gy respectively. According to the Mann-Whitney *U* test, the two techniques showed statistically significant (p < 0.05) differences for all other dose volume histogram parameters as shown in Table 3.

**Table 3:** Treatment planning dosimetric results for the 3DCRT_DIBH and HT_FB plans.

	3DCRT_DIBH (mean ± SD)	HT_FB (mean ± SD)
CTV		
D2% (Gy)	108.11 ± 1.43	104.88 ± 0.78
Dmax (Gy)	112.75 ± 2.52	106.81 ± 0.91
Heart		
Mean (Gy)	5.11 ± 1.55	4.63 ± 0.79
V25 (%)	7.90 ± 3.33	0.88 ± 0.66
V5 (%)	11.55 ± 1.79	22.15 ± 3.87
LAD		
Mean (Gy)	30.83 ± 9.2	9.70 ± 3.1
D2% (Gy)	49.15 ± 0.91	20.28 ± 7.38
Ipsilateral Lung		
Mean (Gy)	17.03 ± 2.53	12.48 ± 1.64
V20 (%)	28.40 ± 5.38	18.64 ± 5.96
V40 (%)	17.10 ± 4.61	2.86 ± 2.13
V5 (%)	43.25 ± 3.64	55.57 ± 3.61
Contralateral Lung		
Mean (Gy)	0.47 ± 0.07	3.08 ± 0.74
V5 (%)	0.22 ± 0.23	12.33 ± 1.47
Contralateral Breast		
Mean (Gy)	0.60 ± 0.20	5.01 ± 0.79

A patient-per-patient comparison, showing large inter-patient variation in heart and lung dose is presented in the supplementary data (Figs. S1 and S2). HT_FB offered a dose reduction to most patients, but increased the dose in a few patients in the cohort (points above the identity line). The results show that patients planned with HT_FB technique generally would receive lower mean doses and lower high dose volumes to the heart, LAD, and ipsilateral lung compared to the 3DCRT_DIBH dose plans. However, the 3DCRT_DIBH plans result in lower low dose volumes to the heart and both lungs, as well as lower mean dose to the contralateral lung and breast, compared to the HT_FB dose plans.

**Fig. 1:**
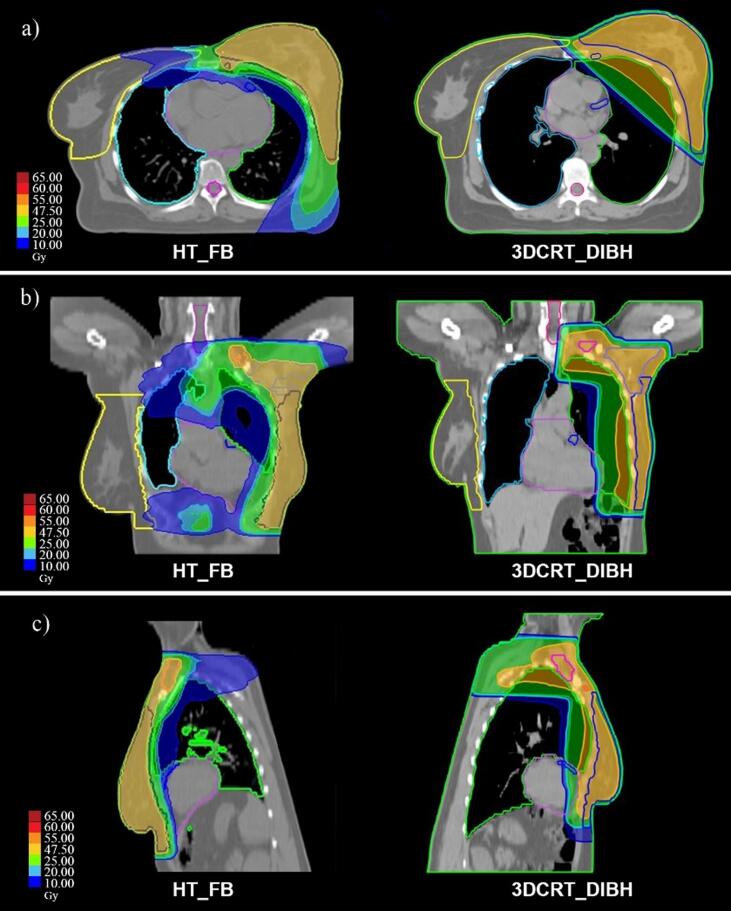
Typical color-wash dose distributions of different treatment plans in axial, coronal, and sagittal planes. HT in FB reduced the ipsilateral lung mean dose, ipsilateral lung V20, heart V25, and LAD mean dose compared with 3DCRT in DIBH. FB: free-breathing; DIBH: deep-inspiration breath-hold; HT: helical tomotherapy; 3DCRT: Three-dimensional conventional radiotherapy.

The dose homogeneity in the breast and lymph nodes were improved for HT_FB inverse planning when compared to forward planning in 3DCRT_DIBH plans with homogeneity index of 1.04 ± 0.0 and 1.08 ± 0.01 respectively. Maximum dose of the CTV was also reported in Table 3. as another surrogate for homogeneity. One major advantage of HT_FB could be the avoidance of breast and lymph nodes fields junction and dose gaps compared to the 3DCRT technique. As a result of the skin-sparing behavior of mega-voltage beams, conventional forward plans are normally cold in superficial regions, while HT_FB with multiple beam angles can increase the dose to the superficial regions by increasing the weight of parts of the beams that are more oblique to the skin (Fig. 1). However, there is a trade-off for increasing the low dose received by the contralateral breast and lung due to the multiple beam angle passing through these organs. Average mean contralateral breast and lung doses were higher for HT_FB (5.01 ± 0.79 Gy and 3.08 ± 0.74 Gy respectively) than with 3D CRT (0.60 ± 0.20 Gy and 0.47 ± 0.07 Gy). The dose received by 2% of the volume of these organs in the medial part was managed to be less than 20 Gy.

Regarding the protocol deviation evaluation for target coverage, there is a concern that 3DCRT planning with almost margin free nodal irradiation at medial part may lead to inadequate coverage of supraclavicular fossa CTV to spare the spinal canal. The heart dose criteria were met for all patients in 3DCRT_DIBH plans. The dose objective of (V20 < 30%) for the ipsilateral lung was met for fifteen out of twenty patients, and V20 was between 30 and 33% for five patients, which shows a maximum violation of about 3% from the protocol. No protocol deviation was observed for target dose coverage or healthy organ sparing in the HT_FB plans.

Regarding the time cost comparison, the planning time for 3DCRT plans was about 2 h for each patient compared to the HT planning of 3 to 4 h as it takes more effort to provide superior target conformality and organ sparing with minimum trade-off via inverse planning. The dose delivery time was almost comparable for 3DCRT_DIBH and HT_FB with an average of about 5 min for each patient. In addition to planning and delivery time, we need to consider about 30 min (in the case of one coaching session) for DIBH training for patients receiving 3DCRT_DIBH. For some patients who need more practice for stability and reproducibility, the workflow consists of several coaching sessions with self-practice at home according to video instruction. Regarding HT_FB, all intensity-modulated plans should be measured before the first treatment fraction according to the National Quality Control program, which adds about 15 min per HT plan. Also, the Quality check (” paper” work) takes longer for an HT plan than for a 3DCRT plan.

## Discussion

Both techniques (HT_FB and 3DCRT_DIBH) kept heart and lung dose below clinical objectives for most left sided breast cancer patients enrolled in this study. Doses to the LAD remained high in selected patients: Mean D2% for the investigated patient group was about 20 Gy in HT_FB, seven patients (with IMN target, challenging anatomy, or tumor bed just in front of the heart) presented high mean D2% (up to 33 Gy) in HT_FB. This is while this value increases up to 49 Gy at 3DCRT_DIBH. Similar results of high values for D2% were published by Poitevin-Chacón *et al*. and Vennarini *et al* [48], [49]. Other studies showed lower heart and LAD mean doses compared with our study, but this may reflect our challenging population, where the majority of the patients are in advanced stages with supraclavicular, axillary, and internal mammary lymph nodes included in radiation fields [13], [50]. While HT_FB inverse planning showed a significant (p < 0.05) reduction in the dose to the heart (except mean dose), LAD and ipsilateral lung compared to 3DCRT_DIBH, this came at the cost of an increased dose to contralateral organs. Therefore, though DIBH is often considered the standard of care in left-sided breast cancer, the selection of the best treatment for each individual patient may be debated amongst clinicians. For example, some oncologists might favor the great conformality and junction-free delivery process of HT_FB for an elderly patient with pre-existing cardiovascular conditions and where sparing the heart may be judged more important than minimizing the risk of second primary cancers. A relatively large dose to the contralateral breast might also make re-treatment more challenging in case of a contralateral recurrence. This is worth considering depending on the patient's age and risk factors.

As reported by literature, the risk of contralateral breast cancer after radiotherapy for breast cancer appears to be highest in women who are younger than age 40–45 years at receipt of radiotherapy [51], [52], [53], [54]. Therefore, 40 years may be considered as an age border for patient selection criteria for intensity modulated techniques. Studies also showed that risks for lung cancer after breast radiotherapy is higher after postmastectomy radiotherapy than other clinical situations [51], [53], [55], [56]. It may provide another patient selection criteria that the complex anatomy of mastectomy patients or bilateral breast cancer including regional lymph node irradiation may get more benefits in lung dose reduction via highly modulated advanced techniques. There is another anatomical feature in patients with funnel-like chests with convex lungs causing larger lung volume in the radiation fields that may lead the clinical decision toward more advanced radiotherapy techniques [57]. Also, in patients not being able to comply with DIBH or not being prioritized due to limited availability in our center, HT_FB might be an acceptable option to minimize heart dose. In this study, a small proportion of patients was not able to comply with DIBH. The current study might be too small to conclude on the percentage of compliant patients in our breast cancer population. However, this study confirms that the limiting factor in expanding DIBH treatments is likely to be the limited availability of DIBH, rather than compliance.

When comparing dose plan parameters between static field treatment under DIBH to modulated treatment under free breathing its worth mentioning that respiration is one of the dominating motions affecting the radiotherapy treatment. During DIBH the residual motion of the target is within few mm, however, during free breathing the motion extent can be much larger. This might cause deviations between the planned and delivered dose distributions in the form of dose blurring and interplay effects [58]. Interplay effects occur only for dynamic treatment techniques such as VMAT or HT, where there is a simultaneous movement of machine parts (multileaf collimators (MLC), gantry and couch) and the target volume, resulting in hotspots and cold spots in the dose distribution. The phenomenon has been confirmed by Moeckly *et al.* for HT breast treatment with the assumption of reproducible regular and rigid body mechanics in respiratory motion [59]. We have however not addressed any interplay effects in this dose planning study.

Another limitation of the current study is the small number of patients enrolled in this study, which did not allow us to make the comparison in subgroups of early-stage/locally advanced patients or targets with or without IMN. Further analysis with enough statistics and less heterogeneous patient characteristics may be of interest for future studies. The next limitation is the risk of contour differences between the two scans (DIBH and FB). To reduce the contour variation, all contours were reviewed by one radiation oncologist expert in breast cancer. As the calculation algorithms and dose calculation grid size are different between the two plans it may also has impact on the accuracy of the dose distribution calculated especially for narrow organs like the LAD. As reported by AAPM Task Group 101, 2 mm grids are required for IMRT procedures, especially in high-dose gradient areas. They also reported that a 2.5 mm isotropic grid produces an accuracy of about 1% in the high-dose region of an IMRT plan consisting of multiple fields [60]. Therefore, grid size of<1 mm in HT can be considered well accurate and we do not expect that a resolution of 3 mm in 3DCRT plans show a clinically significant grid size effect on our comparison. The resolution in cranial-caudal direction is 5 mm for both plans due the CT slice thickness.

Due to limited availability, our institution currently prioritizes younger patients with higher heart doses (mean dose and V25) for DIBH treatment. The results of this study have motivated us to implement breast IMRT (helical tomotherapy) in free breathing as a possible alternative for cardiac and lung sparing in patients who cannot be prioritized for DIBH treatments, especially for plans when the target volumes consist of breast and IMNs**.**

## Conclusion

Although DIBH is considered to be the most effective technique in mean heart and ipsilateral lung dose reductions for left sided breast cancer radiotherapy using 3DCRT, we have showed that helical tomotherapy in free breathing might also be an appropriate alternative treatment technique. Especially for heart, LAD and lung sparing in challenging cases, such as patients with advanced disease or patients who cannot comply with, or cannot be prioritized for, DIBH.

## Declaration of Competing Interest

The authors declare that they have no known competing financial interests or personal relationships that could have appeared to influence the work reported in this paper.
