# Supplementary material for: A dose planning study for cardiac and lung dose sparing techniques in left breast cancer radiotherapy: Can free breathing helical tomotherapy be considered as an alternative for deep inspiration breath hold?
Source: Tech Innov Patient Support Radiat Oncol. 2023 Jan 26;25:100201. doi: 10.1016/j.tipsro.2023.100201 (PMC9926227; doi:10.1016/j.tipsro.2023.100201)
Supplement: Supplementary data 1 [file mmc1.docx]

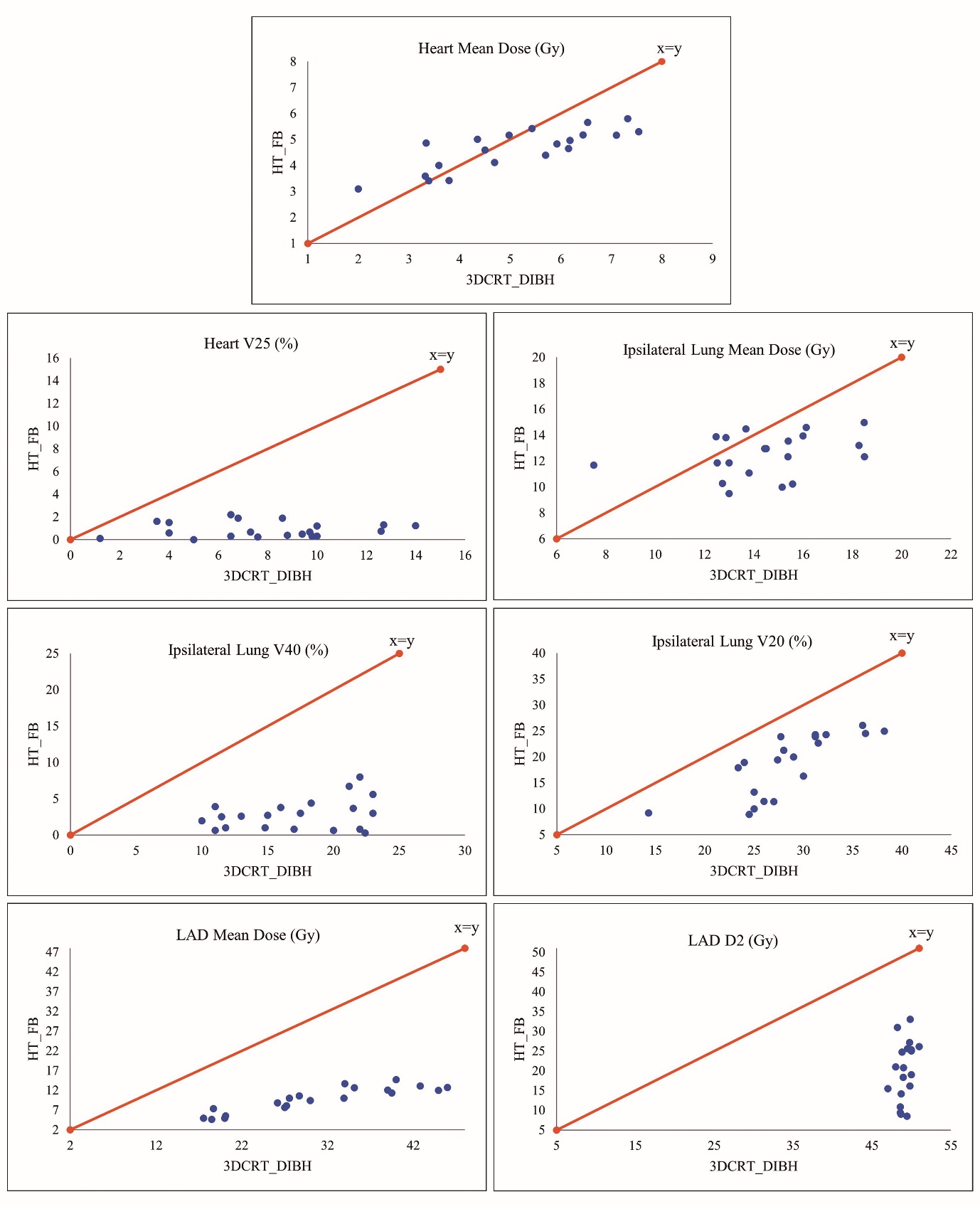
**Supplementary**

Fig. S1. **Benefit HT_FB.** Scatter plots for different dose volume histogram parameters of heart, LAD, and ipsilateral lung. Vx is the volume (%) receiving x dose (Gy) or higher and Dx is the dose (Gy) delivered to the x volume (%) or higher. The identity line of x=y may be used as a reference in comparing two sets of data for two techniques. Points above the identity line of x=y indicated a dosimetric benefit for 3DCRT-DIBH, while points below the line indicate a dosimetric benefit for HT_FB.


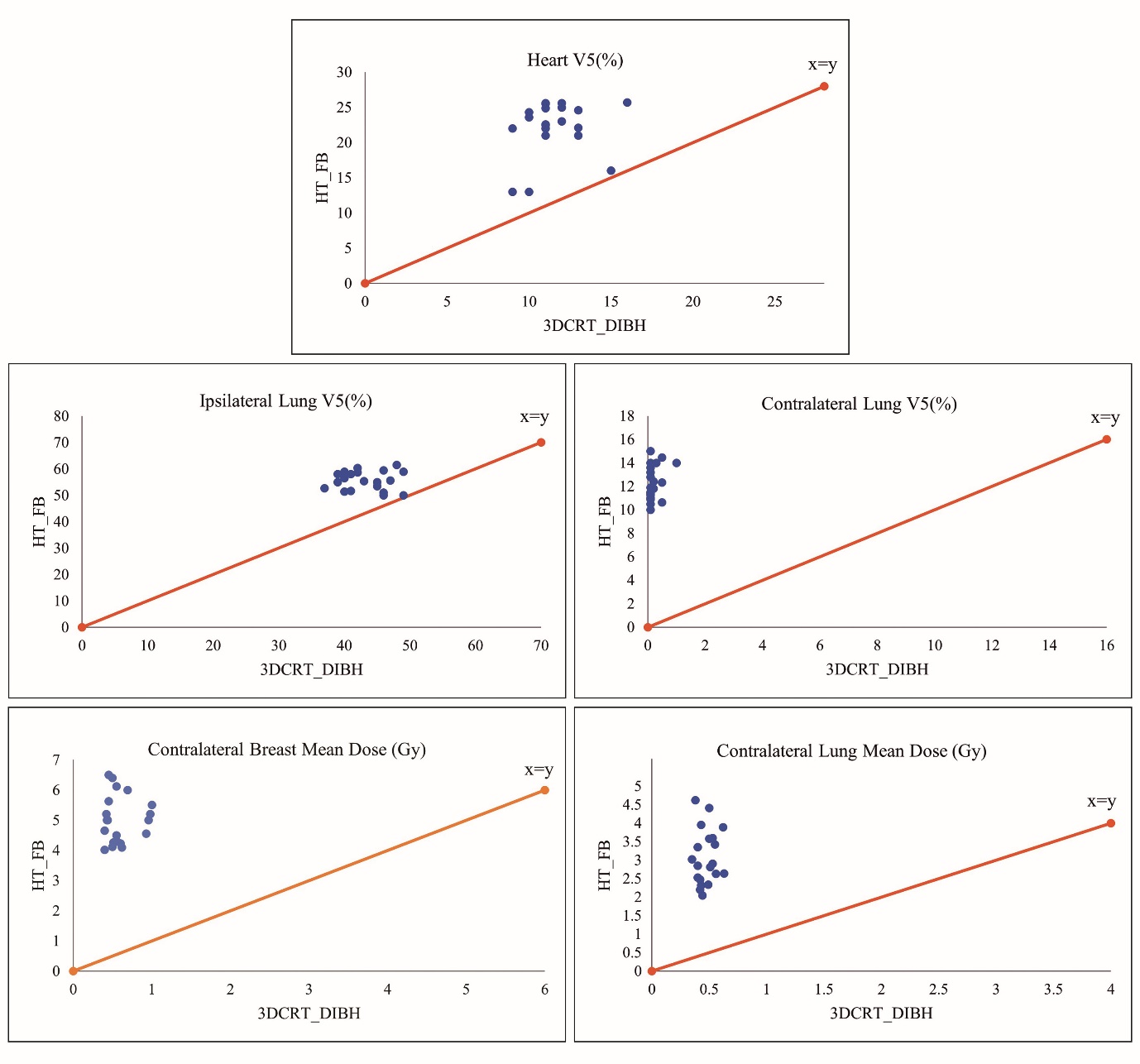


Fig. S2. **Benefit 3DCRT_DIBH.** Patient-specific results for contralateral breast and lung mean dose and V5 for heart and lungs. V5 is the volume (%) receiving 5Gy or higher. Points above the identity line of x=y indicated a dosimetric benefit for 3DCRT-DIBH, while points below the line indicate a dosimetric benefit for HT_FB.
